# The Comorbidity Between Internet Gaming Disorder and Depression: Interrelationship and Neural Mechanisms

**DOI:** 10.3389/fpsyt.2018.00154

**Published:** 2018-04-23

**Authors:** Lu Liu, Yuan-Wei Yao, Chiang-shan R. Li, Jin-Tao Zhang, Cui-Cui Xia, Jing Lan, Shan-Shan Ma, Nan Zhou, Xiao-Yi Fang

**Affiliations:** ^1^Faculty of Psychology, Institute of Developmental Psychology, Beijing Normal University, Beijing, China; ^2^State Key Laboratory of Cognitive Neuroscience and Learning and IDG/McGovern Institute for Brain Research, Beijing Normal University, Beijing, China; ^3^Department of Psychiatry, Yale University School of Medicine, New Haven, CT, United States; ^4^Beijing Huilongguan Hospital, Beijing, China; ^5^Students Counseling Center, Beijing Normal University, Beijing, China

**Keywords:** amygdala, depression, fMRI, internet gaming disorder, resting-state functional connectivity, subgenual anterior cingulate cortex

## Abstract

Internet gaming disorder (IGD) is characterized by cognitive and emotional deficits. Previous studies have reported the co-occurrence of IGD and depression. However, extant brain imaging research has largely focused on cognitive deficits in IGD. Few studies have addressed the comorbidity between IGD and depression symptoms and underlying neural mechanisms. Here, we systematically investigated this issue by combining a longitudinal survey study, a cross-sectional resting-state functional connectivity (rsFC) study and an intervention study. Autoregressive cross-lagged modeling on a longitudinal dataset of college students showed that IGD severity and depression are reciprocally predictive. At the neural level, individuals with IGD exhibited enhanced rsFC between the left amygdala and right dorsolateral prefrontal cortex (DLPFC), inferior frontal and precentral gyrus, compared with control participants, and the amygdala-frontoparietal connectivity at the baseline negatively predicted reduction in depression symptoms following a psychotherapy intervention. Further, following the intervention, individuals with IGD showed decreased connectivity between the left amygdala and left middle frontal and precentral gyrus, as compared with the non-intervention group. These findings together suggest that IGD may be closely associated with depression; aberrant rsFC between emotion and executive control networks may underlie depression and represent a therapeutic target in individuals with IGD.

Registry name: The behavioral and brain mechanism of IGD;

URL: https://www.clinicaltrials.gov/ct2/show/NCT02550405;

Registration number: NCT02550405.

## Introduction

Behavioral addictions and substance use disorders share many clinical manifestations including comorbidities such as depression [1]. Internet addiction (IA) has been regarded as a putative behavioral addiction. Internet gaming disorder (IGD), as a most prevalent form of IA, has been included in the fifth edition of the Diagnostic and Statistical Manual of Mental Disorders (DSM-5) as a condition warranting further study [2]. Psychiatric illnesses have conventionally been considered as categorically distinct entities. However, in the initiative of Research Domain Criteria (RDoC), neurobiological markers of cognitive and emotional dysfunctions are considered to be of significant importance in diagnostic classification and may be shared between neuropsychiatric conditions [3]. In particular, brain imaging has provided an efficient tool in identifying these neural markers. Previous studies examined the neural bases of cognitive impairments such as deficient inhibitory control and maladaptive decision-making in IGD [4, 5]. However, emotional dysfunctions (e.g., depression) and the underlying neural mechanisms in this population remained largely unclear despite high comorbidity of IGD and depression.

Depression symptoms frequently occur in individuals with IA/IGD [6]. A meta-analysis reported a significantly higher proportion of patients with depression in individuals with IA (26.3%) than in healthy controls (11.7%) [7]. Studies in IGD also reported higher depressive tendencies in individuals at risk for or with IGD, as well as reduction in depression during remission from IGD [8–10]. However, these cross-sectional findings could not clarify the directionality between IA/IGD and depression [11, 12]. A prospective study would help further revealing the interrelationship between symptoms of IGD and depression.

Resting-state fMRI has emerged as a widely used tool to investigate intrinsic brain activity [13, 14] and cerebral dysfunction in many neuropsychiatric disorders, including IGD and major depressive disorder (MDD) [15, 16]. Importantly, IGD and MDD appear to share resting-state functional connectivity (rsFC) alterations in the emotional network, comprising the amygdala and subgenual anterior cingulate cortex (sgACC). Specifically, the amygdala contributes to the detection and integration of interceptive and autonomic information and emotional stimuli, and to formation and storage of negative emotion memories [11, 15, 17–19]. The sgACC plays a critical role in regulating arousal in response to emotional and other salient stimuli [20, 21]. Previous studies reported maladaptive interactions of the amygdala with regions of the executive control network, including the lateral prefrontal cortex (PFC), in link with excessive responses to negative stimuli both in MDD [22–24] and IGD [25]. The sgACC is central to affective regulation [15, 22] and the pathogenesis of depression [15, 26]. Interconnected with the sgACC and amygdala, the PFC is part of the task control circuit that regulates emotion [27]. MDD patients showed elevated connectivity between the sgACC and dorsolateral/dorsomedial PFC, in association with excessive self-directed rumination [28, 29]. Increased sgACC-PFC connectivity has also been found in individuals with drug addiction [30, 31]. Thus, examining the functional connectivities between the amygdala, sgACC, and PFC, as well as their relationship with depression and addiction severity may reveal critical neural phenotypes of IGD.

Furthermore, previous studies showed that behavioral interventions are effective in ameliorating both addiction severity [32, 33] and depression symptoms in individuals with IGD or IA in general [34–36]. Examining how behavioral interventions influence emotional network connectivity and its associations with reduction in depression and addiction symptoms would provide additional evidence in support of shared neural substrates of IGD and depression.

In the current study, we presented findings from a 4-year longitudinal survey to explore the interrelationship between symptom severity of depression and addiction in IGD. Furthermore, to elucidate the neural networks underlying depression in individuals with IGD, we conducted a cross-sectional rsFC study focusing on the amygdala and sgACC. Finally, we examined how behavioral treatment ameliorated depression and remediated circuit dysfunction in link with depression in individuals with IGD. Based on previous behavioral evidence [11, 12, 37], we hypothesized a bidirectional relationship between past and future severity of Internet addiction/depression symptoms. Further, based on previous neuropsychiatric studies [25, 38], we hypothesized that individuals with IGD would show depression symptoms and altered rsFC of amygdala and sgACC with regions of the executive control network, which could be alleviated by the behavioral intervention for IGD.

## Materials and methods

### Participants

For Study 1, the data were collected as part of a longitudinal study of college students' Internet use at a university in Beijing, in four waves, starting in year 2011. By means of an online survey tool, a cohort of first-year college students was evaluated yearly. All participants provided written informed consent and were financially compensated for their time, according to a protocol approved by the Institutional Review Board of the School of Psychology, Beijing Normal University.

Survey participants were included in the study only if they had played online games and spent on average over 20% of their daily time using internet for gaming during each of the four consecutive years from which the data were taken. Of a total of 2,182 students, 1,619 (1,253 females, 366 males) did not meet the inclusion criteria and were excluded from the study. The exclusion ratio of females (90.99%) was higher than males (45.47%) (χ^2^ = 550.056, *P* < 0.001). Thus, surveys from a total of 563 students (124 females and 439 males) were obtained for the study. Their age ranged from 16 to 21 years (mean ± *SD* = 18.31 ±.89) at Time 1.

Study 2 and 3 were both parts of a larger project of developing and evaluating a behavioral intervention for IGD. Participants were recruited via the Internet and advertisements posted at local universities, with the following inclusion criteria: (1) a score > 67 on the CIAS [39]; (2) > 14 h per week engaged in Internet gaming, for a minimum of 1 year. Inclusion criteria for healthy control (HC) participants were: (1) a score < 60 on the CIAS; (2) never having spent more than 2 h per week engaged in Internet gaming. All participants were right-handed males. Exclusion criteria were any current or previous use of illegal substances and gambling (including online gambling), any history of psychiatric or neurological illness and current use of psychotropic medications, as assessed by a semi-structured interview. A total of 76 individuals with IGD and 41 HCs participated in Study 2. For Study 3, 63 individuals with IGD were recruited, among which 44 agreed to participate in a craving behavioral intervention (CBI+ group) and the rest 19 were in the control group (CBI− group) because of their work schedule. Twenty three individuals within the CBI+ group participated in resting-state fMRI before and after CBI. Sixteen out of 19 CBI− were similarly scanned at the same time points. Studies 2 and 3 were approved by the Institutional Review Board of the State Key Laboratory of Cognitive Neuroscience and Learning at Beijing Normal University.

### Measures

For Study 1, 2, and 3, we measured the severity of Internet addiction among college gamers using the Chinese Internet Addiction Scale (CIAS; 40), which consists of 26 items on a 4-point Likert scale assessing 5 dimensions of symptoms/consequences including compulsive use, withdrawal, tolerance, and problems of interpersonal relationships and health/time management. The reliability and validity of the CIAS have been demonstrated previously for college students [40], and in the current experiment, the Cronbach's alpha coefficients of this scale were 0.933–0.950 across the four time points. For Study 1, we measured depressive symptoms using the thirteen items from the Symptom Checklist (SCL-90) [41]. These items were rated on a scale of 1 (never true) to 4 (always true). In the current experiment, the Cronbach's alpha coefficients for this scale were 0.888–0.936 across the four time points. In studies 2 and 3, participants' depression symptoms were measured using Beck Depression Inventory (BDI) [42].

### MRI data acquisition

For Studies 2 and 3, MRI data acquisition and preprocessing were described in detail in previous study [33]. Briefly, resting-state fMRI data were obtained on a 3.0 T Siemens Trio scanner at Brain Imaging Center, Beijing Normal University. Parameters for the EPI data were: repetition time = 2,000 ms, echo time = 30 ms, flip angle = 90°, field of view = 200 × 200 mm^2^, acquisition matrix = 64 × 64, voxel size = 3.1 × 3.1 × 3.5 mm^3^, slice = 33, time point = 200. A T1-wighted scan was also acquired with following parameters: repetition time = 2,530 ms, echo time = 3.39 ms, flip angle = 7°, field of view = 256 × 256 mm^2^, voxel size = 1 × 1 × 1.33 mm^3^, slice number = 144.

### Craving behavioral intervention (CBI)

The CBI was developed on the basis of a behavioral intervention developed earlier [33]. Complex psychological processes intertwined with emotional dysfunction [43], craving may play a critical role in the development and maintenance of IGD. Interventions that help individuals cope with and reduce craving may promote positive outcomes and prevent relapse (see the Methods section of Supplementary Materials for further details).

### Statistical analysis

#### Autoregressive cross-lagged modeling

For Study 1, we employed autoregressive cross-lagged modeling (ACLM) to assess the longitudinal and reciprocal relationships between severity of addiction and depressive symptoms. The ACLM is well-suited for examining the relations between two constructs over time. In ACLM, autoregressive parameter represents how well an earlier measure y_t_ predicts the later measure of y_(t+1)_, and the cross-lagged parameter represents how an earlier measure z_t_ predicts a later measure of y_(t+1)_ above and beyond the previous measure of y_t_ [44, 45]. The ACLM has been widely used in investigating the temporal inter-relationships of clinical including addiction symptoms [37, 46, 47]. The set of autoregressive cross-lagged models were tested in Mplus 7.4 [48]. Mplus uses the full information maximum likelihood (FIML) estimation method to handle missing data (see Supplementary Materials for further details). SPSS 20.0 was used for descriptive statistics.

#### Testing invariance across time

The ACLM included eight constructs: depression and addiction severity at Times 1, 2, 3, and 4. At each time point, the CIAS subscales constituted the latent variable of Internet addiction severity, and depression severity was indexed by depression subscale score of the SCL-90. To evaluate the autoregressive and cross-lagged effects, we examined the configural, metric (i.e., loading) and structural invariance sequentially. We compared the model fit indices of four nested models (Table 1).

**Table 1:** Comparison of the autoregressive cross-lagged models.

**Models**	**χ^2^**	***df***	**CFI**	**TLI**	**RMSEA**	**CI**
Model 1	441.049	210	0.972	0.963	0.044	0.038–0.050
Model 2	451.961	222	0.972	0.965	0.043	0.037–0.049
Model 3	456.558	226	0.972	0.965	0.043	0.037–0.048
Model 4	481.169	230	0.969	0.963	0.044	0.039–0.050

*CFI, Comparative Fit Index; TLI, Tucker-Lewis Index; RMSEA, Root Mean Square Error of Approximation*.

**Figure 1:**
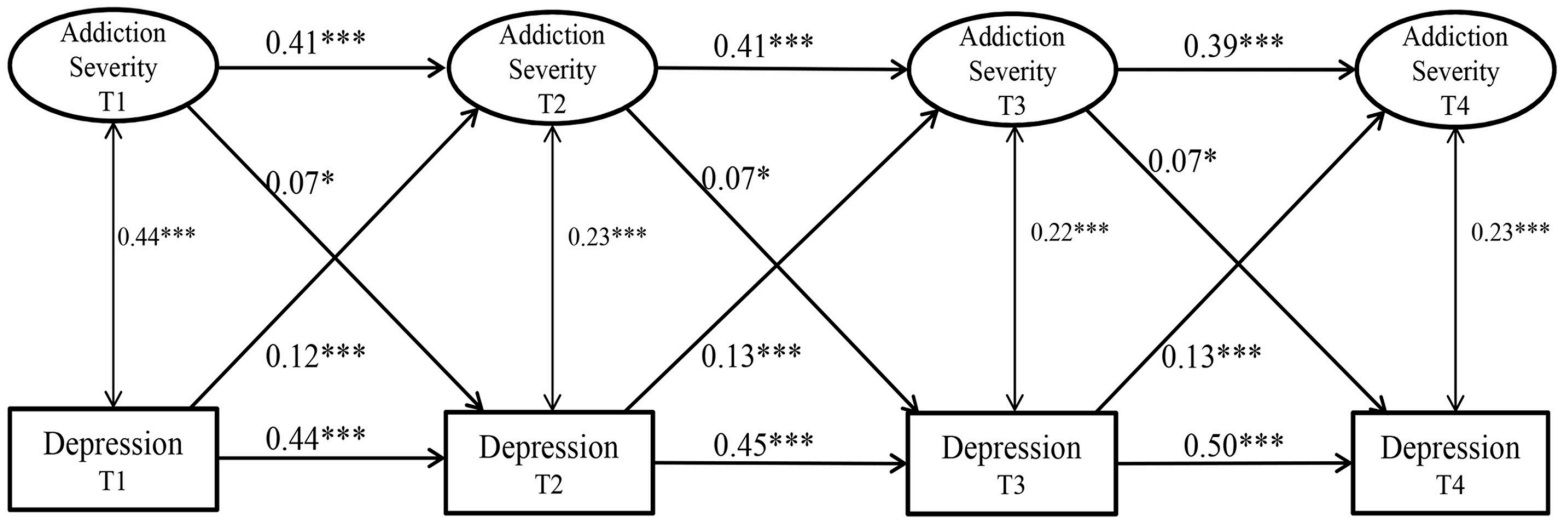
The cross-lagged regression analysis. We indicate the metric invariance, configural invariance, and invariance of error covariance across time using letters on the paths. The numbers are standardized path coefficients (^*^P < 0.05; ^***^P < 0.001).

Model 1 served as the base model with no invariance constraints to test configural invariance. In Model 2, we tested the metric invariance by constraining the factor loadings to be equal across time (Table S2), to ensure that the constructs have the same meaning at each time point [50, 51]. In Model 3, we restricted the cross-lagged paths for depression severity (T) 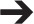 addiction severity (T+1) and addiction severity (T) 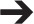 depression severity (T+1) to be equal across time, respectively. Finally, in Model 4, we constrained the auto-regressive paths each for depression and addiction severity across time to be equal (Figure 1). We then compared the model fit indices of all four models sequentially to select the best model. The χ^2^ value, the comparative fit index (CFI), Tucker-Lewis index (TLI) and the root mean square error of approximation (RMSEA) were applied to compare model fit [49].

#### Behavioral data statistical analysis

In Study 2, two sample *t*-tests were conducted to compare addiction and depression severity between the IGD and HC groups. Analyses of variance (ANOVAs) with repeated measures were used in study 3 to examine the effects of CBI on Internet gaming characteristics, with group (CBI + and CBI−) as a between-subject factor, and session (baseline and second test) as a within-subject factor.

#### MRI data preprocessing

Data were preprocessed and analyzed using DPABI version 1.2 (http://rfmri.org/dpabi) and SPM8 (http://www.fil.ion.ucl.ac.uk/spm). The first 10 volumes were discarded. Individual EPI data were slice-time corrected. Participants whose head motion exceeding 3.0 mm in translation or 3° in rotation (2 IGD subjects) were excluded. We further reduced potential confounds of head motion with Friston-24 correction. We regressed out the signals from cerebrospinal fluid and white matter to reduce possible effects of physiological artifacts. EPI data were then normalized to the Montreal Neurological Institute (MNI) space. A spatial filter of 4 mm full width at half maximum Gaussian kernel was used. Subsequently, a band pass temporal filter (0.01–0.10 Hz) was applied to reduce the low-frequency drifts and high-frequency noise.

#### rsFC calculations

Bilateral subgenual ACC and amygdala seeds were identified from a connectivity-based parcellation atlas [52], and from atlas of Brodmann area (Brodmann area 34, see Figure S1). The average time-series within each seed were regressed against whole-brain voxels to generate cross-correlation maps. Correlation coefficients were converted to Z scores with Fisher's r-to-z transform.

We contrasted the IGD and HC groups' rsFC in the sgACC and amygdala for Study 2, and contrasted the rsFC changes between the CBI+ and CBI− groups ([rsFC at the second scanning]−[rsFC at baseline]) in Study 3 with two-sample *t*-tests and the group-difference maps were corrected by means of Gaussian random field theory (GRFT, voxel-level *P* < 0.001 combined with cluster-level *P* < 0.05 corrected for family-wise error).

Within the IGD group in Study 2, we further conducted ROI-based regression analyses to examine the relationships between BDI, CIAS score, and rs-FC, with the ROI's identified from whole-brain between-group comparisons. We reported significant brain activations within the ROIs as corrected by means of GRFT with voxel-level *P* < 0.005 and cluster-level *P* < 0.05 (*P*_*SVC*−*FWE*_ < 0.05).

For Study 3, ROI-based regression analyses were conducted within the CBI+ group to examine the relationships between changes in BDI and in CIAS score and altered rsFC as identified from the two-sample *t*-tests (voxel-level *P* < 0.005 and cluster-level *P* < 0.05; *P*_*SVC*−*FWE*_ < 0.05).

## Results

### Study 1: a longitudinal survey of depression and addiction severity in internet gamers

Bivariate correlations demonstrated moderate stability of the same variables across the four waves, significant concurrent correlations between variables within each wave, and significant longitudinal correlations across waves (see Table S1). Specifically, across the four waves, severity of Internet addiction earlier was associated with higher depression later (*r'*s ranging from 0.19 to 0.27, *P* < 0.01), and higher depression earlier was associated with greater addiction severity later (*r'*s ranging from 0.25 to 0.30, *P* < 0.01).

To test bidirectional relationships between addiction and depression severity, we first fit Model 1 without any covariates or constraints. The model fit for this basic model was good [$\chi_{\left(210\right)}^{2}$ = 441.049, *P* < 0.001, CFI = 0.972, RMSEA = 0.044, SRMR = 0.070]. Model 1 served as the base model for comparison with more constrained models, where each of the cross-lagged paths was constrained to be equal across measurements. Consistent with our hypotheses, Model 2 showed better fit than Model 1 with better RMSEA but no significant difference in χ^2^, CFI and TLI values [Δ$\chi_{\left(12\right)}^{2}$ = 10.912, *P* > 0.05; ΔCFI < 0.01, ΔTLI < 0.01]. Thus, metric invariance of Internet addiction was supported, suggesting that addiction severity was understood and assessed by online gamers to be the same across the 4 years. Second, Model 3 was better compared to Model 2, with slightly better RMSEA but same CFI, TLI and χ^2^ value. That is, the cross-lagged effects of the two relations [depression/addiction severity (T) 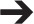 addiction/depression severity (T+1)] were identical across the 4 years. Next, Model 4 differed from Model 3 in χ^2^ but not other fit indices (ΔCFI < 0.01, ΔTLI < 0.01, ΔRMSEA < 0.01), suggesting that each autoregressive effect of the two variables was stable and identical across the 4 years. Model 4 was thus selected as a final model for this study.

Table 2 lists the path coefficients of Model 1 and 4, and shows that the severity of Internet addiction and depression symptoms was positively correlated over time. Furthermore, the impact of depression on addiction severity (β = 0.118, 0.126, 0.127) was higher than the impact of addiction severity on depression (β = 0.070, 0.066, 0.070). Together, these results provide statistical measures of the temporal interrelationship between depression and addiction severity.

**Table 2:** Parameter estimates of the basic model and the ARCL Model 6.

**Paths**	**Basic Model 1**		**Model 4**	
	**B (β)**	**CR**	**B (β)**	**CR**
Severity of addiction (T1) 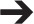 Severity of addiction (T2)	0.429 (0.399)	7.654^***^	0.417 (0.407)	13.555^***^
Severity of addiction (T2) 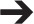 Severity of addiction (T3)	0.452 (0.447)	8.437^***^	0.417 (0.408)	13.555^***^
Severity of addiction (T3) 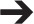 Severity of addiction (T4)	0.355 (0.348)	5.841^***^	0.417 (0.392)	13.555^***^
Depressive symptoms(T1) 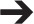 Depressive symptoms(T2)	0.599 (0.537)	12.242^***^	0.481 (0.444)	17.591^***^
Depressive symptoms(T2) 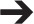 Depressive symptoms(T3)	0.567 (0.514)	11.718^***^	0.481 (0.446)	17.591^***^
Depressive symptoms(T3) 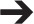 Depressive symptoms(T4)	0.332 (0.391)	7.805^***^	0.481 (0.500)	17.591^***^
Severity of addiction (T1) 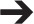 Depressive symptoms(T2)	−0.295 (−0.016)	−0.348	1.237 (0.070)	2.492^*^
Severity of addiction (T2) 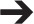 Depressive symptoms(T3)	2.241 (0.117)	2.513^*^	1.237 (0.066)	2.492^*^
Severity of addiction (T3) 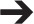 Depressive symptoms(T4)	1.363 (0.084)	1.564	1.237 (0.070)	2.492^*^
Depressive symptoms (T1) 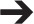 Severity of addiction(T2)	0.009 (0.134)	2.805^**^	0.007 (0.118)	4.456^***^
Depressive symptoms (T2) 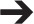 Severity of addiction(T3)	0.008 (0.131)	2.709^**^	0.007 (0.126)	4.456^***^
Depressive symptoms (T3) 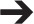 Severity of addiction(T4)	0.007 (0.126)	2.291^*^	0.007 (0.127)	4.456^***^

* P < 0.05;

** P < 0.01;

*** *P < 0.001; T1, Time 1; T2, Time 2; T3, Time 3; T4, Time 4*.

### Study 2: neural correlates of depression in internet gaming disorders

#### Demographics and internet gaming characteristics of IGD and HC subjects

IGD and HC subjects did not differ in age, education, or alcohol use and cigarette smoking measures. As expected, IGD subjects reported higher BDI (8.78 ± 5.54 vs. 2.85 ± 3.64; *t* = 6.91, *P* < 0.001) and higher CIAS scores (78.46 ± 8.40 vs. 43.49 ± 9.64; *t* = 20.27, *P* < 0.001), in comparison to HC subjects (Table S3).

#### rsFC differences between IGD and HC subjects

**Figure 2:**
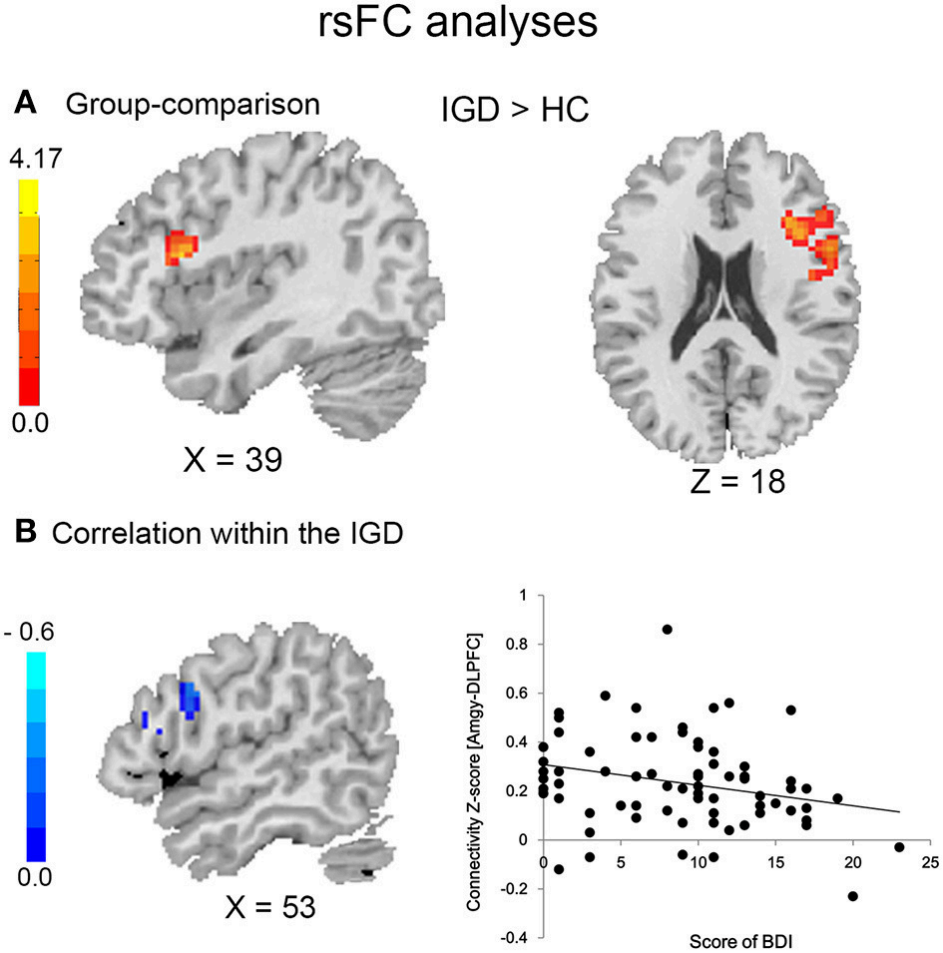
Resting-state functional connectivity in IGD and HC subjects **(A)** and association with depression in IGD group **(B)**.

Compared to HC, IGD subjects showed significantly higher rsFC between the left amygdala and right DLPFC (Figure 2 and Table 3). However, no significant between-group differences were observed for the right amygdala or bilateral sgACC seeds. By using a more liberal criterion (voxel level *P* < 0.005 and cluster-level *P* < 0.05), IGD subjects showed significantly higher rsFC between the left sgACC and right DLPFC (Figure S2 and Table S4).

**Table 3:** Seed locations and regions showing significant differences in connectivity between IGD and HC subjects (GRFT, voxel level *P* < 0.001 and cluster-level *P* < 0.05).

**Seed**	**Region**	**Hemis-phere**	**BA**	**Cluster size**	**Peak MNI (mm)**			**Peak T**
					**X**	**Y**	**Z**	
Left amygdala	DLPFC/IFG/OFC	R	9, 45, 44, 46	284	39 54	18 9	18 24	4.17 4.06

*IGD, Internet gaming disorder; HC, healthy control; DLPFC, dorsolateral prefrontal cortex; IFG, inferior frontal gyrus; OFC, Orbitofrontal cortex*.

#### Brain-behavior relationships

Within the IGD group, depression score was negatively correlated with connectivity between the left amygdala and right DLPFC (MNI: 57, 9, 30; *r* = −0.35; Figure 2). There was no significant correlation between addiction severity and left amygdala—right DLPFC connectivity.

### Study 3: the effects of behavioral intervention on depression and the neural bases of therapeutic efficacy

#### Demographics and internet gaming characteristics

ANOVA with repeated measures showed a group (CBI+ & CBI−) by session (first & second assessments) interaction for the severity of IGD [*F*_(1, 59)_ = 22.62, *P* < 0.001] and BDI score [*F*_(1, 59)_ = 7.89, *P* < 0.01] (Table 4). Compared to the control group, the intervention group showed significant reductions in both CIAS and depression scores after treatment.

**Table 4:** Comparisons of measured variables between the CBI+ and the CBI− group at time-points of before and after intervention.

**Variable**	**Group**	**T1**	**T2**	***F^*a*^***	***F^*b*^***
		***M* ± *SD***	***M* ± *SD***		
Depression	CBI+ (*n* = 44)	33.50 ± 7.99	27.01 ± 5.60	7.89^**^	36.80^***^
	CBI− (*n* = 19)	30.47 ± 4.73	29.46 ± 4.82		0.39
	*T*	1.87	−1.75		
CIAS	CBI+ (*n* = 44)	82.23 ± 9.37	60.55 ± 9.08	22.62^***^	140.54^***^
	CBI− (*n* = 19)	76.58 ± 7.52	70.74 ± 8.29		4.41^*^
	*T*	2.32^*^	−4.19^***^		

* p < 0.05;

** p < 0.01;

*** *p < 0.001; **F**^a^: the effects of group × time points interaction; **F**^b^: the simple effects of time points in each group*.

#### Changes in rsFC in the CBI + and CBI− groups

**Figure 3:**
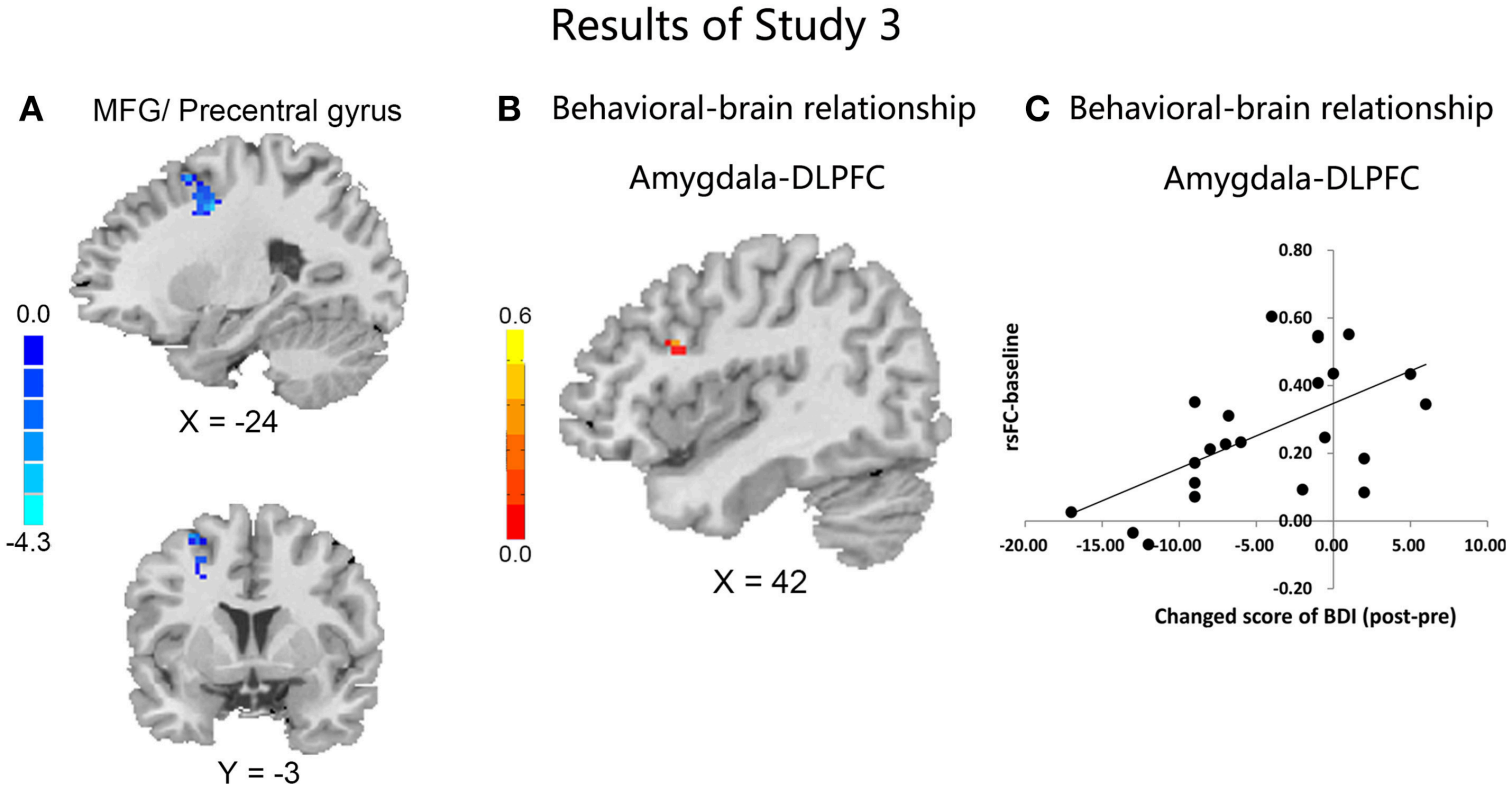
Results in study 3. Comparisons of the rsFC changes ([rsFC at the second scanning]–[rsFC at baseline]) between the CBI+ and CBI− groups over the left amygdala with MFG, precentral gyrus and SFG **(A)**; Negative association between the FC of left amygdala and right DLPFC at baseline with changed score of depression in the CBI+ group **(B)**; Scatterplot is shown of correlation between changed score of BDI and beta values for cluster surviving in baseline rsFC of amygdala-DLPFC **(C)**.

Compared with the CBI− group, the CBI + group showed significantly reduced rsFC of the left amygdala with left precentral gyrus and DLPFC, following the intervention (Figure 3A and Table 5). However, no significant between-group differences were observed for the right amygdala or bilateral sgACC seeds. With a more liberal criterion (voxel level *P* < 0.005 and cluster-level *P* < 0.05), CBI+ subjects showed significantly decreased functional connectivity between the left sgACC and the left postcentral gyrus (Figure S3 and Table S5).

**Table 5:** Seed locations and regions showing significant differences in connectivity between CBI+ and CBI− groups (GRFT, voxel level *P* < 0.001 and cluster-level *P* < 0.05).

**Seed**	**Region**	**Hemis-phere**	**BA**	**Cluster size**	**Peak MNI (mm)**			**Peak T**
					**X**	**Y**	**Z**	
Left amygdala	Precentral gyrus/DLPFC	L	6, 8	44	−24	−3	42	−4.34

*IGD, Internet gaming disorder; HC, healthy control; DLPFC, dorsolateral prefrontal cortex*.

#### Brain-behavior relationships

Although no significant associations between changes of the rsFC and levels of depression or addiction severity were observed in the CBI+ group, the connectivity between left amygdala and right DLPFC at baseline was negatively associated with changed score of depression ([Post-Pre], MNI: 42, 15, 27, *r* = 0.63; SVC; Figures 3B,C) in the CBI+ group. However, the association was no more significant when controlled for the baseline depression severity.

## Discussion

We assessed the relationship between symptoms of depression and addiction and the underpinning neural mechanisms by combining a longitudinal survey study, a cross-sectional resting-state functional connectivity (rsFC) study and an intervention study. In general, Internet addiction and depression maintains a bidirectional relationship among Internet gamers as addiction and depression severities reciprocally influence each other across a 4-year period. By directly comparing individuals with IGD and HC subjects, we found that the IGD group showed higher depression severity and amygdala-DLPFC rsFC, with the strength of the connectivity negatively associated with depression in the IGD group. Furthermore, individuals with IGD showed reduced depression severity and rsFC between the amygdala and DLPFC after receiving a behavioral intervention for IGD. Aberrant interactions between emotional and executive control networks may contribute to depression symptoms in IGD, and interventions targeting these aberrations may alleviate both symptoms of Internet addiction and depression. Together, these findings provide strong support that Internet gaming addiction and depression symptoms are closely interrelated.

The results are consistent with the hypothesis that Internet gamers' symptoms of addiction and depression are reciprocally influenced by each other. Specifically, depression/Internet addiction severity at an earlier time positively predicts addiction/depression severity at a later time point. Thus, addiction and depression severity in online gamers are bi-directionally related, consistent with findings in other addictive disorders [53, 54]. Although previous studies have revealed higher depression among online gamers [5, 16, 55, 56], as well as reciprocal relationships between depression and addiction severity using longitudinal data [57], the current findings are the first to demonstrate a stable bidirectional relationship between symptoms of depression and addiction in Internet gamers. The bidirectional relationship may transpire because (1) individuals cope with their emotional distress by playing Internet games [2, 58]; (2) prolonged Internet gaming induces depression due to lack of or withdrawal from real-life relationships [58, 59]. In addition, some shared factors such as biological, social or early life events may increase the risk both of depression and IGD, as well as their association [58, 60]. Furthermore, the impact of depression on addiction severity appeared to be higher than the impact of addiction on depression, an issue that requires further investigation.

At the neural level, compared with HC, the IGD group showed significantly higher rsFC between the left amygdala and right DLPFC, which was negatively associated with depression severity within the IGD group. The amygdala plays a pivotal role in emotional processing, recognition, and memory formation [11, 17, 19]. Importantly, the amygdala reactivity may be modulated by the PFC, and aberrant neural interaction between these two regions has been characterized in depression. Moreover, the amygdala reactivity may be modulated by the PFC, and aberrant neural interaction between these two regions has been characterized in depression. For example, weaker rsFC between the amygdala and PFC has been demonstrated in previous resting-state studies in depression [23, 24, 61], IGD [25], and alcohol misuse [62]. Decreased PFC-amygdala functional connectivity during emotion-related tasks has also been reported in MDD [27, 38, 63]. The DLPFC supports both cognitive and affective control [64], and altered connectivity between the DLPFC and amygdala may be associated with difficulties or disrupts in negative emotion regulation. In contrast with most previous studies in MDD, the current findings showed elevated amygdala-DLPFC connectivity. An *ad-hoc* explanation is that IGD participants may keep on gaming as a coping strategy to escape from negative emotions [58, 61], engaging the DLPFC in the control of negative emotion, which may be relatively intact in individuals with IGD [65], relative to those with MDD. It should be noted that IGD subjects with higher depression symptoms showed lower connectivity between the amygdala and DLPFC, suggesting that the relationship between depression and amygdala-DLPFC connectivity may be not linear. Thus, IGD subjects with lower depression symptoms may increase prefrontal control over the activity of amygdala to manage emotional problems, but such modulation was not as efficacious or even disrupted in those with more severe depression symptoms. Together, the directionality of the alterations in the amygdala-centered connectivity requires more research, with careful considerations of methodology, severity of depression, functional heterogeneity of prefrontal sub-regions, and the effects of medication treatments.”

Consistent with those from a meta-analysis of behavioral interventions in IGD [34], the current intervention study showed significant reduction in Internet addiction and depression symptoms in the CBI+ group after receiving intervention compared with the CBI− group. Furthermore, the CBI+ group showed reduced rsFC of the amygdala with frontal cortical regions. Thus, CBI seems to normalize the amygdala-DLPFC connectivity by directly reducing the salience of the negative emotional stimuli, so that IGD subjects require less cognitive resources for emotion regulation. Taken together, these findings suggest that functional interactions between the amygdala and DLPFC may serve as a potential neurobiological marker of depression symptoms in IGD and candidate target for clinical interventions.

Contrary to findings from MDD [15, 29, 64], no significant sgACC-centered rsFC alteration was found in individuals with IGD, nor the effect of CBI in remediating rsFC between the sgACC and prefrontal cortex. One possible explanation was that, in study 2 and 3, we excluded IGD subjects with severe depression to control for possible confounding factors, and sgACC dysconnectivity may not manifest in individuals with less severe depression. Another possibility concerns the different mechanisms underlying higher depression symptoms in IGD subjects and MDD patients, an issue to be further investigated by studies of individuals with single and comorbid diagnoses. However, it should be noted that the results showed similar network patterns between the sgACC and amygdala, which was consistent with studies in MDD that aberrant rsFC of affective network overlapped in the prefrontal cortex [23, 29].

The study revealed a bidirectional relationship between depression and addiction severity as well as its underlying neural mechanisms in IGD. At the very least, these findings provide evidence for an important neural phenotype—a potential RDoC [3]—of IGD. These results may also shed new light on the development of more effective interventions for IGD. Emotional dysfunction including depression is regarded as an important therapeutic target in addictions because of its association with relapse [66]. Based on the current findings, depression, and other emotional dysfunction should be taken into account when designing interventions and evaluating therapeutic outcomes for IGD. For example, approaches such as real-time fMRI neuro-feedback [67] to modulate rsFC of the amygdala and sgACC may effectively ameliorate both IGD and depression symptoms and complement other interventions to achieve better outcomes.

Some limitations should be noted. First, Study 1 used the subscale of the SCL-90, whereas Study 2 and 3 used the BDI to measure depression. Although both are widely used assessment tools with good psychometric properties, the findings remained to be confirmed by studies using consistent measurements. Second, IGD is one of the most studied subtypes of IA. However, one should be cautious to generalize these findings to other subtypes of IA (e.g., cybersexual addiction) [68]. Third, the current study focused on young adults. Adolescence is another critical period for the development of both IGD and many emotional problems, including depression [69]. There is an urgent need for future studies to examine the comorbidity between IGD and depression and the underlying neural mechanisms in adolescents. Fourth, the current findings do not clarify the causal relationship between depression and IGD. Double-blind, randomized, placebo-controlled studies using a combination of fMRI and antidepressant drug may directly address this issue. Fifth, in Study 3, IGD subjects were not randomly assigned to the CBI+ and CBI− groups. Thus, we cannot exclude possible confounding factors such as motivation to receive treatment on the current behavioral and imaging findings. Finally, we determined IGD according to CIAS scores and weekly gaming time. However, such symptom-based definition may lack a solid theoretical basis and bear the risk of pathologizing common behaviors [70]. Thus, new diagnostic tools based on an appropriate operational definition of IGD and considering critical exclusive criteria are recommended for future studies.

In conclusion, using a combination of longitudinal survey, fMRI and intervention studies, we reported that symptoms of Internet addiction and depression were highly correlated with reciprocal influences among Internet gamers. Individuals with IGD showed higher amygdala-DLPFC connectivity, which was negatively associated with depression symptoms, and such alterations as well as fronto-cingulate connectivity were decreased following a behavioral intervention for IGD. Together, high depression symptoms and fronto-cingulato-amgydala circuit dysfunction should be taken into account for diagnostic classification of IGD and development of interventions for IGD.

## Author contributions

J-TZ and X-YF were responsible for the study concept and design; LL, C-CX, JL, and S-SM contributed to the intervention practice and data acquisition; Y-WY, LL, J-TZ, and CL assisted with data analysis and interpretation of findings; LL and Y-WY drafted the manuscript. J-TZ, CL, and X-YF provided critical revision of the manuscript for intellectual content. All authors critically reviewed and approved the final version of the manuscript submitted for publication.

### Conflict of interest statement

The authors declare that the research was conducted in the absence of any commercial or financial relationships that could be construed as a potential conflict of interest.
