# Supplementary material for: The Comorbidity Between Internet Gaming Disorder and Depression: Interrelationship and Neural Mechanisms
Source: Front Psychiatry. 2018 Apr 23;9:154. doi: 10.3389/fpsyt.2018.00154 (PMC5924965; doi:10.3389/fpsyt.2018.00154)
Supplement: Supplementary file 1 [file Presentation1.PDF]

# The comorbidity between Internet gaming disorder and depression: causal relationship and neural mechanisms

### Supplementary

## Methods

### Study 1

*Participants*

The present study is based on all respondents in 2011 (N = 563), who completed the CIAS and SCL-90 questionnaires. For the year 2012–2014, response rates to the measurement were 88.81% (N = 500), 79.04% (N = 445), 71.40% (N = 402) respectively.

### Study 3

*Craving Behavioral Intervention (CBI)*

The CBI for Internet gaming disorder (IGD) was developed on the basis of behavioral intervention (Dong and Potenza, 2014), the craving framework of boundary conditions (McCarthy et al., 2010), and the fulfillment of psychological needs for Internet use (Suler, 1999). The CBI was developed to help individuals with IGD to: 1) recognize subjective craving and its relationship with Internet gaming behaviors; 2) reduce craving through ameliorating the salience of gaming-cues and craving-related irrational beliefs, withdrawal symptoms and other negative affects; 3) enhance self-monitoring and control for craving through time management training; and, 4) relieve fulfillment of psychological needs through Internet use and attenuate the relationship between craving and gaming behaviors through coping skill training.

The CBI was given once a week for 6 weeks, conducted by four therapists with similar clinical background with respect to training in behavioral therapy and group therapy. A pair of therapists was randomly assigned to a CBI+ group. Each session included 5 parts in 2.5-3 hours: a warming-up exercise, a discussion about the homework from the last session (except the first session), a main structured activity, a brief summary, and the homework assignment.

The topics for each session focused on: 1) understanding and perceiving subjective craving for Internet gaming, listing gaming-related scenes that might trigger craving, and mindfulness training for gaming-cue-induced craving and tension; 2) recognizing and testing irrational beliefs regarding craving and exploring other possible inferences; 3) detecting emotions following craving and searching effective experience of craving regulation; mindfulness training to experience and relieve craving-related emotions; 4) shifting participants' fulfillment of psychological needs from the Internet to reality and building adaptive relationships with peers; 5) learning time management and skills training for coping with craving; 6) maintaining the effectiveness of the intervention through reviewing and practicing, and setting up adaptive and positive plans for daily life in the future. In addition, mindfulness training was self-administered every time they experienced craving outside the intervention

hours as an assignment.

## Results

**Table S1**

Correlations among main variables (symptoms of addiction and depression) within and across the four waves in study 1.

| | | 1 | 2 | 3 | 4 | 5 | 6 | 7 | 8 |
|---|---|---|---|---|---|---|---|---|---|
| **1** | **IGD T1** | -- | | | | | | | |
| **2** | **IGD T2** | .452** | -- | | | | | | |
| **3** | **IGD T3** | .369** | .459** | -- | | | | | |
| **4** | **IGD T4** | .265** | .349** | .364** | -- | | | | |
| **5** | **Depressive symptoms T1** | .429** | .297** | .283** | .152** | -- | | | |
| **6** | **Depressive symptoms T2** | .245** | .328** | .272** | 0.098 | .520** | -- | | |
| **7** | **Depressive symptoms T3** | .194** | .271** | .332** | .245** | .437** | .532** | -- | |
| **8** | **Depressive symptoms T4** | .173** | .253** | .187** | .312** | .424** | .431** | .407** | -- |
| **Mean** | | 48.23 | 51.60 | 50.86 | 48.22 | 20.01 | 20.72 | 19.88 | 18.83 |
| **SD** | | 11.68 | 12.00 | 12.27 | 12.61 | 6.73 | 7.43 | 8.23 | 7.05 |

SD= standard deviation; T1= Time 1; T2= Time 2; T3= Time 3; T4= Time 4.

**P* < .05; ***P* < .01; ****P* < .001.

**Table S2**

Factor loadings of Internet addiction severity (scores of CIAS) in Model 2-Study 1.

| Paths | Model 2 | |
|---|---|---|
| | **B (β)** | **CR** |
| Tolerance (T1) → Addiction Severity (T1) | 1.000 (0.756) | - |
| Withdrawal (T1)→ Addiction Severity (T1) | 0.970 (0.767) | 37.181*** |
| Compulsive Use (T1)→ Addiction Severity (T1) | 1.100 (0874) | 42.754*** |
| Problems of Interpersonal Relationships & Health (T1)→ Addiction Severity (T1) | 1.112 (0.869) | 42.573*** |
| Time management (T1)→ Addiction Severity (T1) | 1.015 (0.769) | 38.076*** |

| | | |
|---|---|---|
| Tolerance (T2)→ Addiction Severity (T2) | 1.000 (0.781) | - |
| Withdrawal (T2)→ Addiction Severity (T2) | 0.970 (0.795) | 37.181*** |
| Compulsive Use (T2)→ Addiction Severity (T2) | 1.100 (0.862) | 42.754*** |
| Problems of Interpersonal Relationships & Health (T2)→ Addiction Severity (T2) | 1.112 (0.874) | 42.573*** |
| Time management (T2)→ Addiction Severity (T2) | 1.015 (0.782) | 38.076*** |
| Tolerance (T3) → Addiction Severity (T3) | 1.000 (0.819) | - |
| Withdrawal (T3)→ Addiction Severity (T3) | 0.970 (0.807) | 37.181*** |
| Compulsive Use (T3)→ Addiction Severity (T3) | 1.100 (0.896) | 42.754*** |
| Problems of Interpersonal Relationships & Health (T3)→ Addiction Severity (T3) | 1.112 (0.892) | 42.573*** |
| Time management (T3)→ Addiction Severity (T3) | 1.015 (0.848) | 38.076*** |
| Tolerance (T4)→ Addiction Severity (T4) | 1.000 (0.850) | - |
| Withdrawal (T4)→ Addiction Severity (T4) | 0.970 (0.800) | 37.181*** |
| Compulsive Use (T4)→ Addiction Severity (T4) | 1.100 (0.898) | 42.754*** |
| Problems of Interpersonal Relationships & Health (T4)→ Addiction Severity (T4) | 1.112 (0.887) | 42.573*** |
| Time management (T4)→ Addiction Severity (T4) | 1.015 (0.836) | 38.076*** |

**P* < .05; ***P* < .01; ****P* < .001; T1= Time 1; T2= Time 2; T3= Time 3; T4= Time 4.

**Table S3**

Characteristics and clinical assessments of Study 2.

| | IGDs (*n* = 74) | HCs (*n* = 41) | $t$ / $\chi^2$ | *P*(2-tailed) |
|---|---|---|---|---|
| | Mean (SD) | Mean (SD) | value | |
| age | 22.28 (1.98) | 23.02 (2.09) | -1.89 | .06 |
| education | 15.74 (1.84) | 16.32 (1.71) | -1.64 | .10 |
| CIAS | 78.46 (8.40) | 43.49 (9.64) | 20.27*** | .00 |
| BAI | 5.42 (5.43) | 2.61 (3.26) | 3.47** | .00 |
| BDI | 8.78 (5.54) | 2.85 (3.64) | 6.91** | .00 |
| Alcohol use | 57 | 29 | 0.55 | .46 |

| | | | | |
|---|---|---|---|---|
| (at least once per month) | | | | |
| Cigarette use (at least once per month) | 8 | 0 | 4.76 * | .029 |

SD= standard deviation; IGD= Internet gaming disorder; HC= healthy controls; CIAS= Chen Internet addition scale; BAI= Beck Anxiety Inventory; BDI= Beck Depression Inventory.

**Table S4**

Seed location and regions showing significant differences in connectivity between IGD and HC subjects (GRFT, voxel level *P* < 0.005 and cluster-level *P* < 0.05).

| Seed | Region | Hemis-phere | BA | Cluster size | Peak MNI (mm) X | Y | Z | Peak T |
|---|---|---|---|---|---|---|---|---|
| Left sgACC | DLPFC/ MFG/ IFG/ OFC | R | 46, 10, 9, 45, | 420 | 42 | 45 | 3 | 3.64 |
| Left Amygdala | DLPFC/ IFG/ IPL/ STG/ OFC | R/L | 5, 48, 46, 21 | 5452 | 3 | -42 | 66 | 4.30 |

IGD = Internet gaming disorder; HC = healthy control; DLPFC= dorsolateral prefrontal cortex; MFG = middle frontal gyrus; IFG = inferior frontal gyrus; OFC= Orbitofrontal cortex; IPL= inferior parietal lobule; STG= superior temporal gyrus.

**Table 5**

Seed location and regions showing significant differences in connectivity between CBI + and CBI– groups (GRFT, voxel level *P* < 0.005 and cluster-level *P* < 0.05).

| Seed | Region | Hemis-phere | BA | Cluster size | Peak MNI (mm) X | Y | Z | Peak T |
|---|---|---|---|---|---|---|---|---|
| Left sgACC | Postcentral gyrus / Precentral gyrus / IPL/ | L | 4, 40, 2, 3 | 206 | -42 | -21 | 45 | -4.60 |
| Left Amygdala | DLPFC/ MFG/ Precentral gyrus/ SFG/ | L | 8, 6 | 170 | -24 | -3 | 42 | -4.34 |
| | IPL/ Postcentral gyrus/ SMG | L | 40, 3 | 90 | -42 | -42 | 45 | -4.03 |

IGD = Internet gaming disorder; HC = healthy control; IPL = inferior parietal lobule; DLPFC= dorsolateral prefrontal cortex; MFG = middle frontal gyrus; SFG= superior frontal gyrus; SMG= supramarginal gyrus.

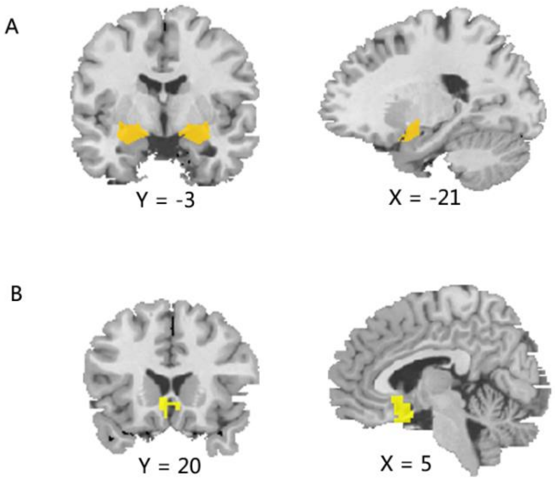


Figure S1. Seed regions of amygdala (A) and sgACC (B) in MNI coordinate.

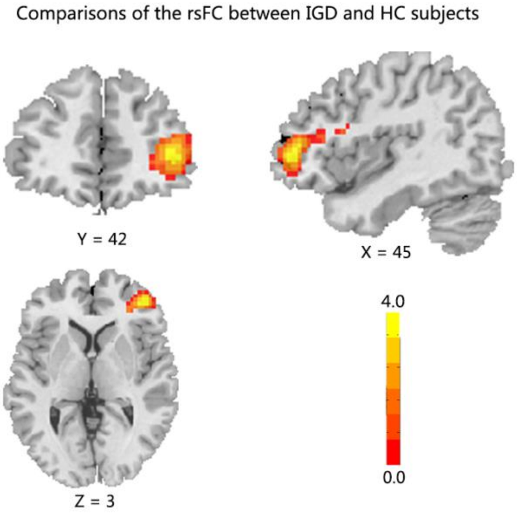


Figure S2. Resting-state functional connectivity in IGD and HC subjects. The left sgACC with DLPFC, MFG, IFG and OFC.

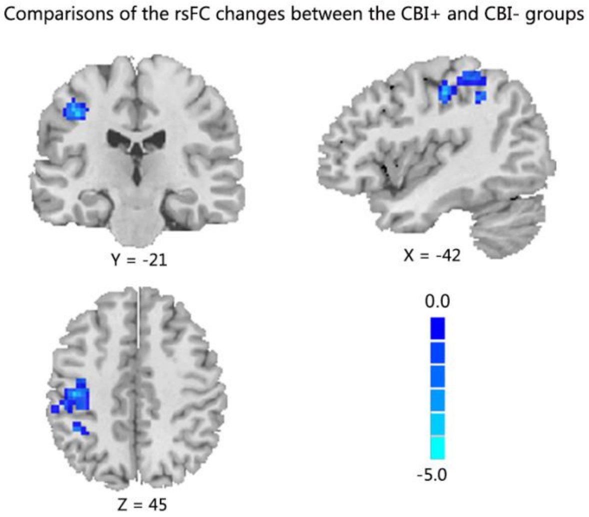

Figure S3. Comparisons of the rsFC changes ([rsFC at the second scanning]–[rsFC at baseline]) between the CBI+ and CBI– groups. The left sgACC with postcentral gyrus and precentral gyrus.
